# Increasing participation in resistance training using outdoor gyms: A study protocol for the ecofit type III hybrid effectiveness implementation trial

**DOI:** 10.1016/j.conctc.2024.101358

**Published:** 2024-08-24

**Authors:** Anna K. Jansson, David R. Lubans, Mitch J. Duncan, Jordan J. Smith, Adrian Bauman, John Attia, Sara L. Robards, Emily R. Cox, Sam Beacroft, Ronald C. Plotnikoff

**Affiliations:** a Centre for Active Living and Learning, School of Education, University of Newcastle, Callaghan, NSW, Australia; b Active Living and Learning Research Program, Hunter Medical Research Institute, New Lambton Heights, NSW, Australia; c Faculty of Sport and Health Sciences, University of Jyväskylä, Jyväskylä, Finland; d School of Medicine and Public Health, University of Newcastle, Callaghan, Australia; e School of Public Health, University of Sydney, Camperdown, NSW, Australia; f School of Biomedical Sciences and Pharmacy, University of Newcastle, NSW, Australia

**Keywords:** Resistance training, Physical activity, mhealth, Outdoor gym

## Abstract

**Background:**

In this paper we outline the protocol for an implementation-effectiveness trial of *ecofit*, a multi-component mHealth intervention aimed at increasing participation in resistance and aerobic physical activity using the outdoor built environment (i.e., outdoor gyms) and social support. We have previously demonstrated the efficacy and effectiveness of the *ecofit* program in insufficiently active people with (or at risk of) type 2 diabetes and community-dwelling adults, respectively. The objective of this trial is to compare the effects of two implementation support models (i.e., ‘Low’ versus ‘Moderate’) on the reach (primary outcome), uptake, dose received, impact and fidelity of the *ecofit* program.

**Research design and methods:**

This hybrid type III implementation-effectiveness study will be evaluated using a two-arm randomized controlled trial, including 16 outdoor gym locations in two large regional municipalities in New South Wales, Australia. Outdoor gym locations will be pair-matched, based on an established socio-economic status consensus-based index (high versus low), and randomized to the ‘Low’ (i.e., *ecofit* app only) or ‘Moderate’ (i.e., *ecofit* app, face-to-face workout sessions and QR codes) implementation support group. The primary outcome of ‘reach’ will be measured using a modified version of the ‘System for Observing Play and Recreation in Communities’, capturing outdoor gym use amongst community members.

**Conclusion:**

This implementation-effectiveness trial will evaluate the effects of different levels of implementation support on participation in resistance-focused physical activity using mHealth and outdoor gyms across the broader community. This may guide widespread dissemination for councils (municipalities) nation-wide wanting to promote outdoor gym usage.

**Trial registry:**

This trial was preregistered with the Australian and New Zealand Clinical Trial Registry (ACTRN12624000261516).

## 1 Introduction

Physical inactivity accounts for 9 % of premature mortality (5.3 million deaths) worldwide and is one of the leading risk factors for non-communicable diseases [1]. In Australia, physical inactivity accounts for 10–20 % of the national disease burden for several major non-communicable diseases (e.g., diabetes, different cancers, dementia, coronary heart disease and stroke) and is responsible for 3 % of the total burden of disease [2]. Insufficient activity results in annual healthcare expenditures of approximately AUS$850 million, and AUS$15.6 billion in productivity loss [3]. National and international guidelines [4] recommend adults engage in 150–300 min of moderate-intensity (or 75–150 min of vigorous-intensity) aerobic physical activity weekly, and resistance training (RT) on at least two days/week. The prevalence of meeting both aerobic and resistance activity guidelines globally and in Australia is 17 % and 15 % for adults, respectively [5], with fewer Australian adults achieving RT (19–40 %) [[6], [7], [8], [9]] than the aerobic physical activity guidelines (45 %) [8]. Despite the consequences of inactivity and the many health-related benefits [[10], [11], [12], [13], [14], [15], [16]] of meeting both physical activity guidelines [4], few at-scale physical activity interventions have been conducted to improve participation rates [17]. To enhance population physical activity behaviours and health-related outcomes, effective interventions that are ‘scalable’ are needed [18].

Within public health, ‘scale-up’ refers to the “*… deliberate efforts to increase the impact of successfully tested health interventions so as to benefit more people and to foster policy and program development on a lasting basis*” [19]. Some aerobic-based physical activity interventions targeting adults have been scaled-up from efficacy to effectiveness to dissemination, becoming embedded in systems outside of the research setting (e.g., the 10,000 Steps program [[20], [21], [22]]). By comparison, few RT interventions targeting adults have progressed from efficacy to effectiveness [23,24], and to our knowledge, no interventions have been disseminated and implemented on a larger scale. While examples of scaled-up RT interventions exist in school settings (e.g., ‘Resistance Training for Teens’ [25]), the lack of scale-up trials targeting adults is concerning, given the higher proportion of not meeting the RT guidelines [[6], [7], [8]]. It may be that interventions promoting aerobic exercise are easier and cheaper to scale-up compared with those involving RT, as most RT interventions are supervised, include specialised equipment (e.g., weight machines, free weights, elastic resistance bands), are delivered within exercise facilities, and require a different sets of skills (e.g., correct lifting techniques versus going for a walk) [26,27]. Knowledge of the independent health benefits of RT is also more recent than for aerobic activities, providing another potential explanation for this research/practice gap. Regardless, the presence of explicit recommendations to participate in RT warrants a focus on this important, yet often overlooked health behaviour [28].

**Fig. 1:**
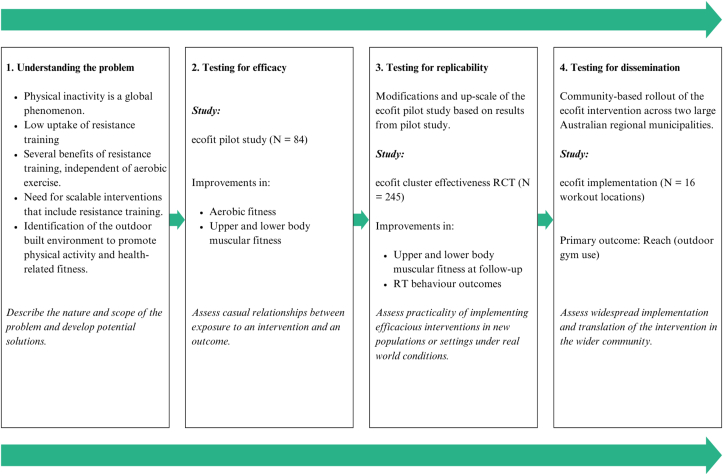
Research translational continuum of the ecofit intervention.

*ecofit* is a community-based, multi-component intervention that promotes resistance and aerobic physical activity using smartphone technology, the outdoor built environment and social support. The *ecofit* intervention has been evaluated in an efficacy trial targeting adults at risk of/diagnosed with type 2 diabetes, and in an effectiveness trial targeting community-dwelling adults failing to satisfy physical activity recommendations. The efficacy trial showed significant improvements in both primary outcomes (i.e., aerobic fitness and lower body muscular fitness) at the primary time-point, while the effectiveness trial showed significant improvements in upper and lower body muscular fitness at follow-up only [29,30]. For a more detailed account of the efficacy and effectiveness trials, see the published protocols [31,32]. This research protocol will outline the steps guiding the translation pathway of *ecofit* to larger scale implementation [33,34]. Fig. 1 provides a summary of the translation pathway for the ecofit intervention. This will be done by testing a ‘Low’ versus a ‘Moderate’ implementation support model on the *ecofit* intervention in two large regional municipalities in New South Wales, Australia, on the reach (primary outcome), uptake, dose received, impact and fidelity. The planning and design have been guided by the Medical Research Council recommendations for developing and evaluating complex interventions [35] and the Consolidated Framework for Implementation Research (CFIR) [36].

## 2 Materials and methods

### 2.1 Experimental design

We will evaluate the implementation of *ecofit* using a hybrid type III implementation-effectiveness trial [37] involving 16 outdoor gym locations randomized to either the (i) ‘Low’ or (ii) ‘Moderate’ implementation support model. Assessments will be conducted at baseline, 3-months (primary and secondary outcomes) and 6-months (secondary outcomes only) and will commence in mid-2024. This implementation-effectiveness trial will be conducted across all outdoor gym locations (N = 16) in the Newcastle City (population n = 173,251) and Lake Macquarie (population n = 202,680) local government municipalities, New South Wales (NSW), Australia. The Standards for Reporting Implementation Studies (StaRI) initiative will be used in the reporting of this study [38]. Our study has received ethical approval from the University of Newcastle Ethics committee (H-2018-0060). The trial has been registered with the Australian and New Zealand Clinical Trial Registry (ANZCTR) (ACTRN12624000261516).

### 2.2 Recruitment, randomization and blinding

We have developed a promotional campaign to promote outdoor gym use across both local government municipalities; this will serve as the primary recruitment strategy for the study. The campaign will employ strategies, used in the *ecofit* effectiveness trial [31] and other, similar studies [39], including print and broadcast media, letter drops to all households within the same or adjacent suburbs of each outdoor gym, school/work newsletters, and social media, to promote the *ecofit* app and outdoor gym use. The promotional information will also emphasise the benefits of RT.

Randomization of the park location has already been conducted. Each outdoor gym was pair-matched based on the socioeconomic status of the area surrounding the facilities (high versus low). This classification was estimated using an established socioeconomic status consensus-based index (i.e., the SEIFA index of relative socio-economic advantage and disadvantage) [40]. Parks within each pair were randomized to one of the two conditions (i.e*.,* ‘Low’ or ‘Moderate’ implementation support). The randomization procedure was conducted by a researcher not part of the core research team. The randomization outcome for each location can be found in supplementary material 1. Data collection will be blinded to group allocation.

### 2.3 Outdoor gym quality audit tool

As part of this study, an audit was conducted to determine the ‘quality’ of the outdoor gym facilities by a single researcher. Our audit tool was developed for this study, as there were no existing validated instruments for assessing the quality of outdoor gyms. Items include: (i) general cleanliness of equipment (ii) presence of substantial rust on gym equipment, (iii) broken/damaged equipment (percentage of available equipment), (iv) safety issues surrounding equipment, (v) instructional signage or pre-existing QR codes to online instructions (i.e., not QR codes created for this program), (vi) accessibility (i.e., parking within 100 m of facilities), (vii) accessibility (i.e., quality walking paths leading to equipment, (vii) presence of security cameras, and (ix) lighting around the outdoor gym. The items are assessed via ‘yes’ (definitely present), ‘somewhat’ (partly present) and ‘no/none’ (not present). The majority of outdoor gyms were judged as clean with good access and did not have substantial rust or safety hazards related to the outdoor gym area (e.g., broken equipment, sharp edges, fallen branches). Most had existing support on how to use the equipment, lighting was poor in most locations and there were no security cameras present. The audit instrument and results are included in supplementary material 2.

### 2.4 Intervention components, delivery, and implementation strategies

**Fig. 2:**
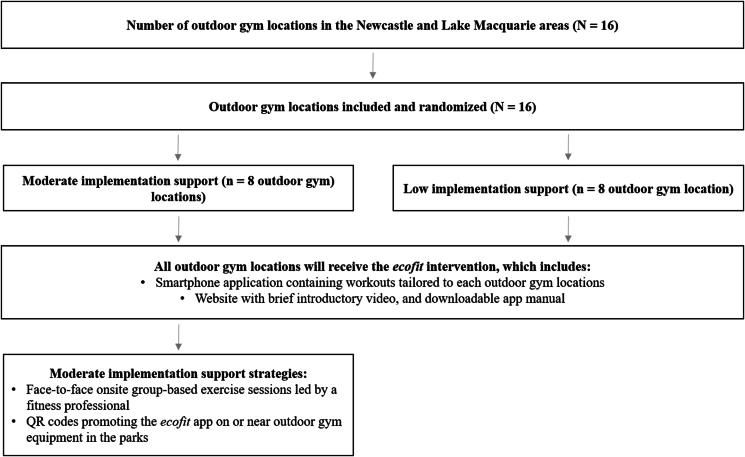
Outline of the Low and Moderate implementation support models promoting the ecofit intervention.

This study will assess the implementation-effectiveness of two different implementation support models promoting the ecofit intervention, which are outlined in Fig. 2 (i) Low support (i.e., *ecofit* app): versus (ii) Moderate support (i.e., *ecofit* app, face-to-face workout sessions and QR codes). These implementation support strategies were guided by the findings from our previous studies [30,31,41,42]. See Table 1 for an overview of the two main previous studies.

**Table 1:** Overview of the efficacy and effectiveness trials and key changes.

Efficacy trial design (N = 84) [30,32]	Key changes made for the effectiveness trial	Effectiveness trial design (N = 245) [29,31]	Key changes made for the implementation-effectiveness trial
*Target population:* Adults at risk of, or diagnosed with, type 2 diabetes. *Design:* Two-arm RCT evaluating a 20-week community-based physical activity intervention integrating outdoor built environment (e.g., some outdoor gym equipment, park benches, trees, railings), smartphone technology (i.e., app), and social support (i.e., face-to-face workout and cognitive mentoring sessions). *Intervention:* The intervention group was provided a smartphone application (app), which included workouts and cognitive challenges. In the first 10-weeks of the intervention, participants also received five face-to-face workout/skill-building and psycho-educational sessions. *Primary outcomes:* Aerobic fitness and lower body muscular fitness. *Results:* Significant improvements in aerobic and lower body muscular fitness at the primary time-point (10-wk) and follow-up (20-wk).	• Partnership with Newcastle and Lake Macquarie City Councils. • More inclusive eligibility criteria • Larger sample size. • Removed face-to-face workout and cognitive mentoring sessions. • Developed a more comprehensive smartphone application to substitute the face-to-face component. • Only provided participants with one introductory session and an app manual. • Workouts had more focus on using the outdoor gym equipment to perform exercises. • Primary outcomes targeting muscular fitness only.	*Target population:* Adults not meeting the aerobic and/or the resistance training guidelines. *Design:* Two-arm cluster RCT evaluating a 9-month community-based mHealth physical activity intervention (strong focus on RT), which prescribed workouts using locally available outdoor gym facilities through a smartphone application. To promote social support, participants were able to enrol as groups (of up to 4) and join the *ecofit* Facebook group. *Intervention:* The intervention group was provided with access to a smartphone app, which included standardised workouts tailored to each workout location. In addition, participants had to attend a 90-min introduction session prior to receiving access to the app. *Primary outcomes:* Upper and lower body muscular fitness. *Results:* Significant improvements in upper and lower body muscular fitness at follow-up (9-months), but not at the primary time-point (3-months).	• Shifting focus from individual health-related outcomes to reach and uptake. • Inclusion of more outdoor gym locations (due to recent installations). • Reintroducing face-to-face sessions at the intervention parks. Although, these will not include the psychology-based component. • Intervention parks will also include QR codes to download the app. • Adding a website with introductory videos to the app and a downloadable app manual. • Removing the happiness-item and the 12-min walk/run test from the self-assessment.

### 2.5 Intervention components

#### 2.5.1 ecofit app

The *ecofit* application has been updated based on feedback from the smartphone app used in the effectiveness trial [29,31] and the need to increase the scalability of its functions from a design perspective. The new *ecofit* platform can be accessed both via a smartphone app downloadable for apple and android devices, as well as via website browsers. The functionality has been improved and expanded upon compared with the previous app [31], and now includes a more scalable approach to tailoring workouts.

**Fig. 3:**
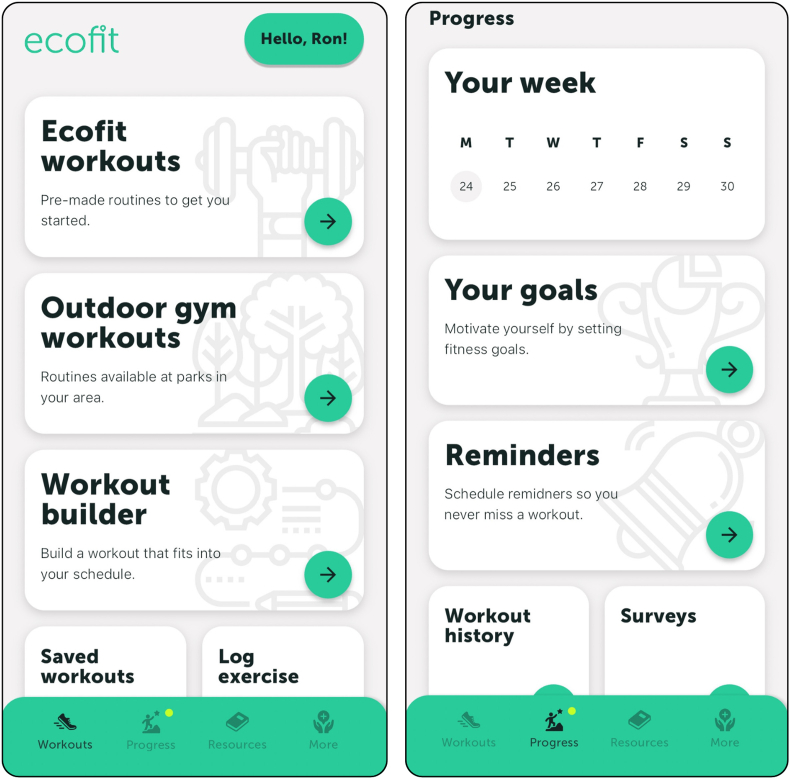
Screenshots of the home page and progress page from the ecofit app.

The new platform includes (i) videos with real people demonstrating the exercises in an outdoor gym or home-based setting, (ii) written instructions of each exercise alongside the videos, (iii) four different difficulty levels (i.e., level 1 to 4), (iv) ability to choose predetermined or ‘create your own’ workouts, (v) self-monitoring functions (i.e., goal setting and self-assessment tool), (vi) an exercise library, and (vii) resources based on behaviour change techniques [43]. Instead of a face-to-face introductory session, as was used in the effectiveness trial [31], the new *ecofit* platform includes a built-in function that guides users through the app upon registration. Alongside this, users will also have access to an introductory video explaining the purpose of *ecofit* and an onboarding video, which provides an overview of how to use the *ecofit* app. The app will be available to download free of charge, with no exclusion criteria. Informed consent will be collected via the app upon registration. See Fig. 3 for a screenshot of the *ecofit* app homepage and ‘progress’ section.

#### 2.5.2 ecofit workouts

The workouts on the *ecofit* platform have been expanded upon to suit a broader range of fitness levels. Adaptations include adding more exercises to the overall library, as well as creating another (lower) level of difficulty which targets individuals with low fitness and/or who may have compromised mobility. The difficulty levels for all exercises range from ‘1’ (easiest) to ‘4’ (most difficult) and were determined by an Accredited Exercise Physiologist. The number of sets for each exercise within the workouts range from 1 to 2 (level 1) to 2–3 (levels 2–4) and the number of recommended repetitions is 10 for each set. Upon program commencement, participants will be instructed to initially complete one set of each exercise. When they are able to complete the full set comfortably, they will be encouraged to complete an additional set. When participants can comfortably complete the maximum recommended number of sets per level, they will be instructed to move to the next level.

**Fig. 4:**
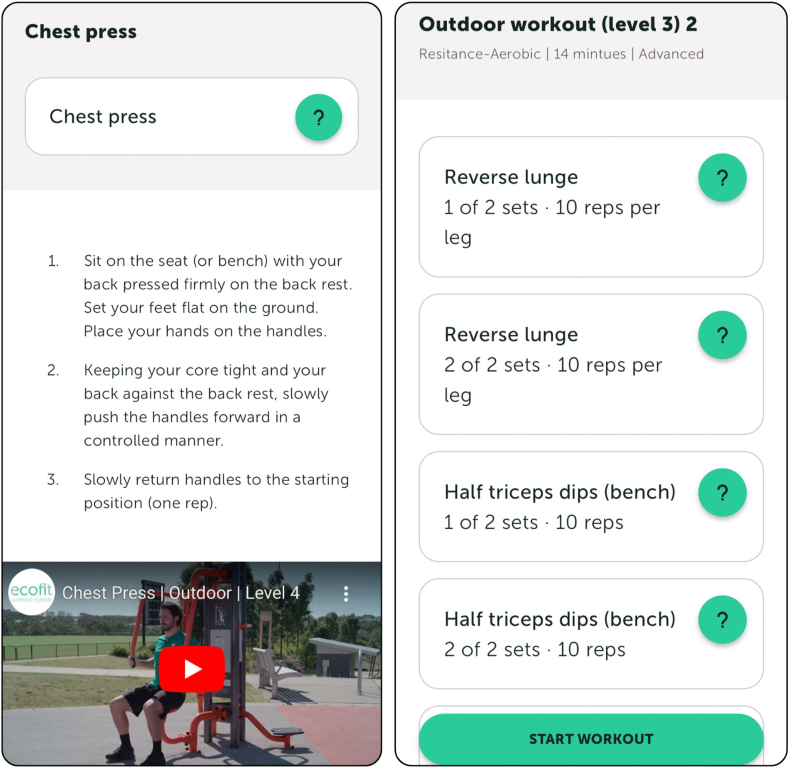
Screenshot of an ecofit workout and instructions of how to perform a specific exercise.

To support scalability, the new *ecofit* platform uses a multi-component approach to creating workouts. These improvements were based on the results from the process evaluation of the effectiveness trial [44], and meetings between researchers and the app design team. App users can choose a ‘generic workout’ (i.e., includes exercises with minimal equipment that can be performed in a home or park-based setting). For park-based settings, the generic workout assumes that there is a wall, bench and railing available, whereas for the home-based setting, it assumes that there is a bench/chair, wall, and light weights (e.g., food cans, water bottles) available. App users can also choose a workout based on specific locations created by the research team (i.e., the research team has mapped out a workout based on the availability of equipment/facilities available at each outdoor gym included in the study) and/or create their own workout by entering the type of equipment available (i.e., the app will generate a workout based on availability of equipment). App users will have the option to choose a workout that targets a specific area of the body (e.g., full body, upper body with/without core, lower body with/without core or core only). See Fig. 4 for a screenshot on an example ecofit workout and instructions to perform a specific exercise.

#### 2.5.3 Outdoor gyms

All outdoor gym locations (regardless of randomization outcome) will be included on the *ecofit* platform. The characteristics (i.e., type, pieces of equipment, socio-economic status of surrounding suburb) of the outdoor gyms are described in Supplementary material 1. The number of pieces of equipment range from three to 19, and the outdoor gym locations are classified as either ‘trail’, ‘pod’ or ‘trail/pod’, as per an earlier published definition [41]. ‘Trail-based’ outdoor gyms consist of ≥2 exercise stations placed sequentially along an existing walking path, where each station includes ≤3 unique pieces of exercise equipment, or equipment intended for performing ≤3 distinct exercises. ‘Pod-based’ outdoor gyms comprise of one station with ≥4 unique pieces of exercise equipment, or equipment intended for performing ≥4 distinct exercises, clustered to one location. ‘Trail/pod-based’ outdoor gyms utilise a combination of trail- and pod-based (at least one pod), which enable users to engage with the exercise equipment either as part of their transit along the trail or solely within the pod station.

### 2.6 Implementation strategies

#### 2.6.1 Face-to-face training sessions

Three, 1-h face-to-face, on-site, group-based exercise sessions, led by a trained fitness professional, will be implemented as part of the Moderate implementation support model. The sessions will be conducted within the first month of the intervention, on set times and days (i.e., weekdays and weekends), and individuals can register to attend the sessions free of charge. Each session will encompass a complete standardised resistance and aerobic-based workout, which has been tailored to the outdoor gym location (i.e., workouts will vary pending availability of equipment). Workouts will follow the same structure as in the *ecofit* app (i.e., 3 x upper body, 3 x lower body and 2 x core exercises, and each exercise will be completed as 2 sets of 10 repetitions) [45]. The aerobic component will vary depending on outdoor gym type (i.e., trail, pod, or trail/pod). For outdoor gym pods, the aerobic activity may include short bursts of aerobic activities between each resistance activity (e.g., star jumps, 100-m walk/run out of/back to the pod, marching/running on the spot) or a longer walk/run after completing the resistance-based exercises. The trail locations will provide the opportunity for aerobic activity when moving between equipment. In addition to the workout, the group-based sessions provide an opportunity to ask questions about the *ecofit* app and receive feedback on their exercise technique. To advertise the face-to-face training sessions, posters will be placed on or near the outdoor gym equipment where the workout sessions will be held, along with schools, shopping centres and community notice boards in suburbs surrounding the outdoor gym locations. Exercise sessions will also be advertised via email to those app users who indicate living within the same postcode of the outdoor gyms allocated to Moderate implementation support model, during the app registration.

#### 2.6.2 Quick-response (QR) codes

QR codes will be placed next to or near the outdoor gyms included in the locations receiving Moderate implementation support. Scanning the QR code will automatically redirect park visitors to the *ecofit* website and provide information about how to download the app. In recent years, QR codes are becoming increasingly popular to use in various research fields and non-research settings [46]. Websites that include program information and resources have been used in other larger scale physical activity interventions [22].

### 2.7 Outcomes/measures and data collection

Based on an evaluation roadmap for scaling-up physical activity and behavioural nutrition interventions [47], a primary outcome (i.e., reach), and several secondary implementation outcomes (i.e., uptake, dose received, impact, fidelity), secondary intervention outcomes (i.e., app usage, acceptability, feasibility and dose satisfaction) and implementation determinants (acceptability, feasibility and satisfaction of face to face sessions and overall cost effectiveness) will be assessed. The primary implementation outcome of ‘reach’ will be measured at baseline and 3-months (primary timepoint). The secondary implementation and intervention outcomes will be measured at baseline, 3-months (primary timepoint) and 6-months (follow-up). Secondary implementation determinants will be assessed following the face-to-face sessions (acceptability, feasibility and satisfaction of face-to-face sessions) and at the conclusion (overall cost-effectiveness) of the study.

#### 2.7.1 Primary implementation outcome/measures

##### 2.7.1.1 Reach

The primary outcome ‘reach’ is defined as the difference in the absolute number (3-month minus baseline assessments) of outdoor gym users (i.e., people using the outdoor gyms for RT activities) between the two implementation models. This will be measured using a ‘modified version’ of the *System for Observing Play and Recreation in Communities* (SOPARC Resistance Training (hereafter referred to as SOPARC-RT)) [41].

SOPARC-RT has been specifically adapted to capture additional data (not included in the original SOPARC protocol [48]) by coding the interaction with the outdoor gym equipment (i.e., ‘using equipment for RT,’ ‘completing RT without equipment,’ ‘stretching using equipment’ and ‘using equipment for aerobic exercise’) [41]. Each park will have one predetermined target area (i.e., either a pod or one station along a trail) which has been identified for observations during the outdoor gym audit. If the outdoor gym location is classified as a pod/trail, the pod will be selected for observation. At all target areas, one trained research assistant will conduct the observations from a position where the outdoor gym equipment is clearly visible. SOPARC-RT will employ similar recording procedures for ‘jogging tracks’ as per the original SOPARC protocol [48] for all parks regardless of outdoor gym type, and each observation period (shift) will result in 2 h-long observations, as opposed to systematic scans as used in SOPARC. The research assistant observing the target area will code each individual by gender (i.e., woman, man, unsure), and perceived age category (i.e., adult [18–59 years], or senior [60 years or older]). The SOPARC-RT observation tool is attached in supplementary material 3.

The observation schedule will cover two days per park (i.e., one weekday and one weekend day). Each day will be divided into three observation shifts: morning (0600–0800 on weekdays, 0630–0830 on weekends), midday (1200–1400), and evening (1600–1800) [41,48]. Cancelled shifts due to inclement weather (e.g., actual or threat of heavy rain and/or thunderstorm) will be replaced on the following day or on the same day of the subsequent week if weather remains inclement [41,48]. Baseline and follow-up (3-months) observations will be conducted by a team of research assistants over a four-week period at baseline, and 3-months. Pair-matched parks will be observed on the same days. Conducting observations on different days and times over a set period of time is a typical approach for outdoor park observational studies monitoring physical activity [49]. Each park will be assessed over two days with three observation periods per day, totalling six observation periods at baseline and six observation periods at follow-up (3-months). The total number of people observed across these six observation periods will be summed separately for the baseline and follow-up assessments.

#### 2.7.2 Secondary implementation outcomes/measures

##### 2.7.2.1 Dose received

‘Dose received (exposure)’ will also be assessed by the number of logged workouts completed amongst participants, assessed at three- and six-months. Data relating to additional characteristics of ‘dose received (exposure)’ will also be evaluated at three- and six-months, including assessment of (i) changes in the workout difficulty time-period by individuals, (ii) whether app users engage in different workout types, training categories or chose workouts targeting specific body parts, (iv) number of goals set and met, (v) and setting where the workouts were undertaken (i.e., home versus park-based). ‘Dose received (exposure)’ is also operationalized as the proportion of participants logging at least one workout in the app, which will be assessed at three- and six-months.

##### 2.7.2.2 Uptake

‘Uptake’ will be defined as the total number of app registrations at three- and six-months post baseline. Only app registrations from the Newcastle and Lake Macquarie municipalities (i.e., based on postcode) and from the launch date of the promotional campaign will be included in the analysis. ‘Uptake’ will also be evaluated by the number of people attending the face-to-face workout sessions in the parks receiving Moderate implementation support by the three-month time-point. The fitness professionals conducting the park-based face-to-face workout sessions will collect count data on attendees, including perceived gender and age categories.

##### 2.7.2.3 Fidelity of face-to-face delivery

A member of the research team will randomly observe each fitness professional on at least two occasions to assess how the individual conducted the workout sessions at the *ecofit* parks receiving Moderate implementation support. Adherence to the exercise protocol by the fitness professional will be measured as ‘yes’ or ‘no’ by the research team member.

#### 2.7.3 Secondary intervention outcomes/measures

##### 2.7.3.1 Usage

‘Usage’ will be evaluated by the number of views of the resource material (i.e., videos and text) across the *ecofit* platform at three- and six months.

##### 2.7.3.2 Impact

‘Impact’ outcomes will be collected via the built-in self-assessment feature in the *ecofit* app at baseline, three and six-months. App notification will be sent at these time points to encourage users to conduct and enter their own self-assessed upper (i.e., push ups on toes or knees) and lower body (i.e., 60-sec sit-to-stand test) muscular fitness. The push-up and 60-sec sit-to-stand tests have demonstrated strong and moderate validity in a sample of community dwelling adults, respectively [50]. Impact is also considered to be a component of study effectiveness.

##### 2.7.3.3 Acceptability, feasibility, and dose satisfaction of the app

Acceptability of the app will be evaluated using a process evaluation survey through the app (see supplementary material 4). Measures will include satisfaction with the content (i.e., exercises, workouts, resources) and app usability. Users will be prompted to complete the survey 6-weeks after registering an account, only data for users who have engaged with the app will be included.

#### 2.7.4 Secondary implementation determinants

##### 2.7.4.1 Acceptability, feasibility, and dose satisfaction of face-to-face sessions

All fitness professionals will be interviewed at the conclusion of all face-to-face training sessions; perceptions about acceptability, feasibility and satisfaction of delivering the training sessions will be explored. Acceptability and satisfaction will also be evaluated with a follow-up survey among participants who have engaged in at least one face-to-face workout session. Participants will complete these surveys directly following the face-face sessions by scanning a QR code and completing online. See supplementary material 5 for survey.

##### 2.7.4.2 Cost-effectiveness

We will conduct a cost-effectiveness analysis by calculating the resources spent on design, adaption and implementation of the two implementation strategies. The outcomes of reach and uptake will be considered in the analyses.

### 2.8 Data management plans

All data will be entered into a database using unique study codes and stored securely on a password protected computer. Only a limited group of researchers will have access to the database. If any important protocol modifications occur during this study, these will be updated on the Australian and New Zealand Clinical Trial Registry and the journal of publication. Before publication of the primary and secondary outcomes of this study, a deidentified dataset will be uploaded to an appropriate data repository.

### 2.9 Statistical analysis

Statistical analyses of the primary (i.e., reach) and secondary (except for fidelity) outcomes will be conducted with generalised linear models adjusted for the baseline value of the outcome using IBM SPSS Statistics version 30 (IBM Corporation, Armonk, NY) for Windows computers. These models will include fixed effect for study group, baseline value of the outcome, and stratification variables (i.e., high versus low SEIFA value). We will explore descriptive statistics and conduct a moderator analysis on the moderate implementation support strategies (i.e. face to face sessions, QR codes at park) to determine their independent impact on our primary outcome of park use. Additional exploratory sub-group analyses of the intervention effect on the primary (i.e., reach) and secondary outcomes (i.e., uptake, dose received, usage and impact) will be conducted using generalised linear models adjusted for the baseline value of the outcome (i.e., group-by-moderator) by gender (i.e., woman, man, unsure) and age (i.e., adults [18–59 years], and seniors [60 years or older]). Descriptive statistics will be used to examine the other secondary outcome of the study (i.e., fidelity) and the implementation determinants (i.e., acceptability, feasibility, dose satisfaction and cost). Sensitivity analyses will examine the robustness of the findings considering the quality of the outdoor gym facilities (based on the audit tool). Analysis of app usage data will include descriptive statistics and Cox Regression to examine time to non-usage attrition as in prior studies [51,52].

To detect a Cohen's d of 1 (strong effect), we estimate that ∼2 individuals will be observed using the outdoor gyms as intended per day per park (17 individuals in total in each group per day) assuming 80 % power and p = 0.05. Taking clustering by park into consideration and assuming an ICC of 0.05, then the design effect is 1.05, resulting in ∼3 individuals per day/park (24 individuals in total in each intervention group, total n = 48). From our previous work, this number is reasonable to expect [39,41].

## 3 Discussion

This protocol describes the *ecofit* implementation-effectiveness trial, which has been adapted from the *ecofit* efficacy and effectiveness trials [29,30]. *Ecofit* is a multi-component community-based intervention aimed at increasing participation in resistance and aerobic-based physical activity using smartphone technology, the outdoor built environment, and social support. The objective of our current trial is to test a ‘Low’ versus a ‘Moderate’ implementation support model of the *ecofit* intervention in two large regional municipalities in New South Wales, Australia, on the reach (primary outcome), uptake, dose received, impact and fidelity. The main innovation of this study is to test whether an mHealth intervention with additional implementation strategies can increase outdoor gym use in the community [41].

*ecofit* represents a novel and scalable approach for the promotion of RT using outdoor gyms and mHealth. RT interventions targeting the general population are scarce, as most studies to date have predominantly focused on clinical populations using supervised modes of delivery [[53], [54], [55], [56], [57], [58], [59]]. Outdoor gyms provide an opportune delivery setting, as facilities are being rapidly installed around the globe (including Australia), offering free and accessible workout apparatus to perform physical activities (particularly RT) [60]. Despite the large number of installations, the facilities are generally poorly used, and few interventions have been trialled to increase usage [41,60]. The *ecofit* app provides a potentially cost-effective tool for local government agencies to promote the use of the facilities. If the additional support strategies (i.e., app only or inclusion of face-to-face introductory sessions and QR codes promoting app downloads) are deemed effective, they will provide councils with additional approaches to increase outdoor gym use. We plan to share our study findings with our local council partners and other local government agencies.

Scaling-up interventions does not come without challenges. As interventions progress from highly controlled settings (i.e., efficacy trials) towards dissemination in real world conditions, challenges that may arise include difficulties with community recruitment, and maintaining and sustaining programs [61,62]. Scaled-up interventions delivered in more ‘real-world’ contexts generally have smaller effects than their corresponding pre-scale-up trials [63] and the combination of ‘program drift’ and ‘voltage drop’ may limit the ultimate progress toward intervention sustainability and population impact [64]. A potential challenge that may arise during our study includes long periods of inclement weather. However, if the weather impedes outdoor activities, users can access indoor workout options in the *ecofit* app. While the modified SOPARC will be conducted for three 2-h time -periods across one weekday and one weekend day, it will not be able to capture any usage outside of these time-periods. Including longer surveillance of the outdoor gyms is not possible due to financial constraint; this is a limitation of the current study. Finally, given the nature of being a mHealth intervention, technological challenges with the smartphone app may also arise. The app, however, includes a ‘feedback and question’ section, allowing consumers to directly notify the researchers should they experience any challenges.

## 4 Conclusion

This paper has outlined the rationale and study protocol for the *ecofit* implementation trial, which will be implemented across 16 outdoor gym locations in two large regional municipalities, Australia. This implementation-effectiveness trial provides a novel protocol, that is scalable, to evaluate a resistance-focused physical activity intervention using mHealth and outdoor gyms across the broader community.

## Funding

This work is supported by the 10.13039/501100000925National Health and Medical Research Council of Australia (Grant number: APP1134914).

## Data availability

No datasets were generated or analysed during the current study. All relevant data from this study will be made available upon study completion.

## CRediT authorship contribution statement

**Anna K. Jansson:** Writing – review & editing, Writing – original draft, Software, Resources, Methodology, Conceptualization. **David R. Lubans:** Writing – review & editing, Software, Resources, Methodology, Funding acquisition, Conceptualization. **Mitch J. Duncan:** Writing – review & editing, Writing – original draft, Software, Resources, Methodology, Funding acquisition, Conceptualization. **Jordan J. Smith:** Writing – review & editing, Writing – original draft, Resources, Methodology, Funding acquisition, Conceptualization. **Adrian Bauman:** Writing – review & editing, Writing – original draft, Methodology, Funding acquisition, Conceptualization. **John Attia:** Writing – review & editing, Writing – original draft, Resources, Methodology, Funding acquisition, Conceptualization. **Sara L. Robards:** Writing – review & editing, Resources, Methodology, Conceptualization. **Emily R. Cox:** Writing – review & editing, Resources. **Sam Beacroft:** Writing – review & editing, Methodology, Conceptualization. **Ronald C. Plotnikoff:** Writing – review & editing, Writing – original draft, Resources, Methodology, Investigation, Funding acquisition, Conceptualization.

## Declaration of competing interest

The authors declare that they have no known competing financial interests or personal relationships that could have appeared to influence the work reported in this paper.

## Data Availability

No data was used for the research described in the article.
