# Supplementary material for: Increasing participation in resistance training using outdoor gyms: A study protocol for the ecofit type III hybrid effectiveness implementation trial
Source: Contemp Clin Trials Commun. 2024 Aug 24;41:101358. doi: 10.1016/j.conctc.2024.101358 (PMC11399599; doi:10.1016/j.conctc.2024.101358)
Supplement: Multimedia component 2 [file mmc2.docx]

**Outdoor gym audit tool**

**Location:**

|  | 0 | 1 | 2 |
| --- | --- | --- | --- |
| Is the equipment clean? | No/none/limited | Somewhat  25%-75% | Yes/A lot |
| Presence of substantial rust | No/none/limited | Somewhat  25%-75% | Yes/A lot |
| Is there broken/damaged equipment that impacts equipment usability? | No/none/limited | Somewhat  25%-75% | Yes/A lot |
| Are there safety hazards? E.g. Broken equipment, sharp edges, fallen branches, potholes etc. | No/none/limited | Somewhat  25%-75% | Yes/A lot |
| Existing instructional signage/QR codes | No/none/limited | Somewhat  25%-75% | Yes/A lot |
| Parking within 100m. If a trail, is parking close to the start of the trail? | No/none/limited | Somewhat  25%-75% | Yes/A lot |
| Are there quality walking paths accessing the outdoor gym? If a trail, are there good quality paths? | No/none/limited | Somewhat  25%-75% | Yes/A lot |
| Security cameras | No/none/limited | Somewhat  25%-75% | Yes/A lot |
| Is there purpose lighting for the park? | No/none/limited | Somewhat  25%-75% | Yes/A lot |

All parks, n = 16

| Variable | Implementation group | Proportion (%) | | |
| --- | --- | --- | --- | --- |
|  |  | No | Somewhat | Yes |
| Cleanliness of equipment | Moderate | 0 | 12.5 | 87.5 |
|  | Low | 0 | 0 | 100 |
|  | Total | 0 | 6.3 | 93.8 |
| Rust present on equipment | Moderate | 87.5 | 12.5 | 0 |
|  | Low | 100 | 0 | 0 |
|  | Total | 93.8 | 6.3 | 0 |
| Damaged equipment | Moderate | 100 | 0 | 0 |
|  | Low | 100 | 0 | 0 |
|  | Total | 100 | 0 | 0 |
| Hazards surrounding/within outdoor gym | Moderate | 100 | 0 | 0 |
|  | Low | 100 | 0 | 0 |
|  | Total | 100 | 0 | 0 |
| Instructional signage on equipment | Moderate | 25 | 12.5 | 62.5 |
|  | Low | 12.5 | 12.5 | 75 |
|  | Total | 18.8 | 12.5 | 68.8 |
| Parking close to equipment | Moderate | 0 | 12.5 | 87.5 |
|  | Low | 12.5 | 0 | 87.5 |
|  | Total | 6.3 | 6.3 | 87.5 |
| Path leading to equipment | Moderate | 0 | 0 | 100 |
|  | Low | 0 | 0 | 100 |
|  | Total | 0 | 0 | 100 |
| Security cameras present | Moderate | 100 | 0 | 0 |
|  | Low | 100 | 0 | 0 |
|  | Total | 100 | 0 | 0 |
| Lights surrounding area | Moderate | 87.25 | 12.5 | 0 |
|  | Low | 50 | 12.5 | 37.5 |
|  | Total | 68.8 | 12.5 | 18.8 |

Parks randomized to ‘Low’ implementation group (n = 8)

Parks randomized to ‘Moderate’ implementation group (n = 8)

| Variable | Implementation group | Proportion (%) | | |
| --- | --- | --- | --- | --- |
|  |  | No | Somewhat | Yes |
| Cleanliness of equipment | Moderate | 0 | 12.5 | 87.5 |
|  | Low | 0 | 0 | 100 |
| Rust present on equipment | Moderate | 87.5 | 12.5 | 0 |
|  | Low | 100 | 0 | 0 |
| Damaged equipment | Moderate | 100 | 0 | 0 |
|  | Low | 100 | 0 | 0 |
| Hazards surrounding/within outdoor gym | Moderate | 100 | 0 | 0 |
|  | Low | 100 | 0 | 0 |
| Instructional signage on equipment | Moderate | 25 | 12.5 | 62.5 |
|  | Low | 12.5 | 12.5 | 75 |
| Parking close to equipment | Moderate | 0 | 12.5 | 87.5 |
|  | Low | 12.5 | 0 | 87.5 |
| Path leading to equipment | Moderate | 0 | 0 | 100 |
|  | Low | 0 | 0 | 100 |
| Security cameras present | Moderate | 100 | 0 | 0 |
|  | Low | 100 | 0 | 0 |
| Lights surrounding area | Moderate | 87.25 | 12.5 | 0 |
|  | Low | 50 | 12.5 | 37.5 |
