# Supplementary material for: Increasing participation in resistance training using outdoor gyms: A study protocol for the ecofit type III hybrid effectiveness implementation trial
Source: Contemp Clin Trials Commun. 2024 Aug 24;41:101358. doi: 10.1016/j.conctc.2024.101358 (PMC11399599; doi:10.1016/j.conctc.2024.101358)
Supplement: Multimedia component 3 [file mmc3.pdf]

AGE: Adults 18-59 years (A); Seniors 60+ years (S)GENDER: Male (M); Female (F); Unsure (u)

Park name:	
Time (circle):	6.00 AM – 8.00 AM (Weekday) / 6.30 AM – 8.30 AM (Weekend) 12 PM – 2 PM 4 PM – 6 PM
Day and Date:	

OBSERVATION RECORDING SHEET RECORDER (insert name):________________________________

Participant (Notes if needed)	Gender	Age	Outdoor gym interaction			
			In the observation area for intended RT		Stretching against/on equipment	Using the elliptical/bike for Aerobic only
			Using the equipment	Doing RT without the equipment (e.g., squats, lunges, etc)		
1						
2						
3						
4						
5						
6						
7						
8						
9						
10						
11						
12						
13						
14						
15						
16						
17						

REMEMBER: Do not double-count people. If they return to the observation area again and continue to workout in the area don't count as another participant, simply update their first entry if required.

AGE: Adults 18-59 years (A); Seniors 60+ years (S)GENDER: Male (M); Female (F); Unsure (u)

OBSERVATION RECORDING SHEET RECORDER (insert name):____

Participant (Notes if needed)	Gender	Age	Outdoor gym interaction			
			In the observation area for intended RT		Stretching against/on equipment	Using the elliptical/bike for Aerobic only
			Using the equipment	Doing RT without the equipment (e.g., squats, lunges, etc)		
18						
19						
20						
21						
22						
23						
24						
25						
26						
27						
28						
29						
30						
31						
32						
33						
34						

REMEMBER: Do not double-count people. If they return to the observation area again and continue to workout in the area don’t count as another participant, simply update their first entry if required.
