# Supplementary material for: Increasing participation in resistance training using outdoor gyms: A study protocol for the ecofit type III hybrid effectiveness implementation trial
Source: Contemp Clin Trials Commun. 2024 Aug 24;41:101358. doi: 10.1016/j.conctc.2024.101358 (PMC11399599; doi:10.1016/j.conctc.2024.101358)
Supplement: Multimedia component 4 [file mmc4.docx]

| 1. I am satisfied with the *ecofit* app | SD | D | N | A | SA |
| --- | --- | --- | --- | --- | --- |
| 1. The app was easy to navigate | SD | D | N | A | SA |
| 1. The information in the app provided me with enough details to perform muscle strengthening activities using outdoor gyms | SD | D | N | A | SA |
| 1. The ecofit app motivated me to participate in more exercise using outdoor gyms. | SD | D | N | A | SA |
| 1. The information in the app increased my confidence to to exercise using outdoor gyms | SD | D | N | A | SA |
| 1. I intend to use the ecofit app in the future to workout using outdoor gyms | SD | D | N | A | SA |
