# Supplementary material for: Increasing participation in resistance training using outdoor gyms: A study protocol for the ecofit type III hybrid effectiveness implementation trial
Source: Contemp Clin Trials Commun. 2024 Aug 24;41:101358. doi: 10.1016/j.conctc.2024.101358 (PMC11399599; doi:10.1016/j.conctc.2024.101358)
Supplement: Multimedia component 5 [file mmc5.docx]

**Face to face survey – attendees**

Date:

Location:

| 1. I was satisfied with the ecofit face-to face session. | SD | D | N | A | SA |
| --- | --- | --- | --- | --- | --- |
| 1. My confidence to complete resistance training activities has improved because of the face-to-face session. | SD | D | N | A | SA |
| 1. The face-to-face session helped me to improve my resistance training technique. | SD | D | N | A | SA |
| 1. My instructor was knowledgeable about resistance training and physical activity. | SD | D | N | A | SA |
| 1. The instructor was able to answer questions I had. | SD | D | N | A | SA |
| 1. The session provided me with the skills and confidence to complete individual ecofit workouts. | SD | D | N | A | SA |
| 1. The session was appropriate for my needs. | SD | D | N | A | SA |
| 1. I have a better understanding of how to use the ecofit app. | SD | D | N | A | SA |
